# *Chlamydia trachomatis* Biovar L2 Infection in Women in South Africa

**DOI:** 10.3201/eid2311.170758

**Published:** 2017-11

**Authors:** Remco P.H. Peters, Ronan Doyle, Mathys J. Redelinghuys, James A. McIntyre, Georges M. Verjans, Judith Breuer, Marleen M. Kock

**Affiliations:** University of Pretoria, Pretoria, South Africa (R.P.H. Peters, M.J. Redelinghuys, M.M. Kock):; Maastricht University Medical Centre, Maastricht, the Netherlands (R.P.H. Peters);; Anova Health Institute, Johannesburg, South Africa (R.P.H. Peters, J.A. McIntyre);; University College London, London, United Kingdom (R. Doyle, J. Breuer);; University of Cape Town, Cape Town, South Africa (J.A. McIntyre);; Erasmus Medical Centre, Rotterdam, the Netherlands (G.M. Verjans);; National Health Laboratory Service, Pretoria (M.M. Kock)

**Keywords:** *Chlamydia trachomatis*, bacteria, lymphogranuloma venereum, serovar L, biovar L2, genital infection, sexually transmitted infections, South Africa

## Abstract

We detected *Chlamydia trachomatis* biovar L2 in vaginal swab specimens of 7 women with vaginal discharge in South Africa. Whole-genome sequencing directly from clinical specimens identified a closely related cluster of strains. The clinical role of this infection in the context of syndromic management should be clarified.

Infection with *Chlamydia trachomatis* biovar L is known as lymphogranuloma venereum (LGV). This infection usually presents as genital ulcers, followed by an invasion of the lymphatic system resulting in buboes, painful swelling of lymph nodes (*1*). In the past 2 decades, another manifestation of LGV has emerged in North America and Europe: rectal LGV infection causing proctocolitis among men who have sex with men (MSM) (*1*). In this population, urethral LGV also occurs (*2*).

There have been only sporadic reports of rectal and genital LGV infection in women living in the industrialized world (*3*,*4*). Cross-sectional studies from France, Switzerland, and the Netherlands did not detect biovar L in specimens from women with genital or rectal *C. trachomatis* infection (*1*,*5**–**7*). Because lymphatic manifestation has become relatively rare, LGV infection is considered an outbreak mainly among MSM in Europe and North America (*1*). Lymphatic LGV is endemic to Africa, but before our study, it was unknown whether *C. trachomatis* biovar L infections occurred in women in Africa. Thus, we determined the prevalence of this infection in South Africa. 

To determine whether genital *C. trachomatis* biovar L infections occur in women living in South Africa, we analyzed 82 DNA samples extracted from vaginal swab specimens that were positive by a molecular detection assay for *C. trachomatis* infection at the Department of Medical Microbiology at the University of Pretoria. The Faculty of Health Sciences Research Ethics Committee at the University of Pretoria approved the studies in which these specimens were collected. These swab specimens had been collected during 2012–2016 from women attending different healthcare settings: a mobile health clinic in rural Mopani District (n = 52) and 3 departments at the academic hospital in Pretoria: obstetrics and gynecology clinic (n = 14), antiretroviral treatment clinic (n = 10), and sexually transmitted infection (STI) clinic (n = 6). We assessed the presence of LGV in these genital specimens by using specific PCRs for *C. trachomatis* serovar L and serovar L2b (*8*). For positive PCR results, we confirmed the diagnosis by conducting whole-genome sequencing (WGS) of *C. trachomatis* directly from the clinical specimen as described elsewhere (*9*).

Whereas *C. trachomatis* biovar L–specific PCR showed positive results for 7 specimens obtained from women at the antiretroviral treatment (n = 5) and STI (n = 2) clinics in Pretoria, we did not detect LGV in any of the 52 specimens from women in Mopani District. All PCR test results for serovar L2b were negative. The 7 women with genital LGV all had vaginal discharge and were co-infected with another STI (Table).

**Table:** Characteristics of 7 women with vaginal discharge and a positive PCR result for *Chlamydia trachomatis* biovar L, Pretoria, South Africa, 2012–2016*

Patient ID	Healthcare setting	HIV status	Co-infection	*C. trachomatis *WGS result	Mean read depth	Genome coverage, %
1	ART clinic	Positive	*Trichomonas vaginalis*	L2 confirmed	41	99.5
2	ART clinic	Positive	*T. vaginalis*	L2 confirmed	12	98.3
3	ART clinic	Positive	*Mycoplasma genitalium*	L2 confirmed	21	98.6
4	ART clinic	Positive	*M.a genitalium*	L2 confirmed	72	99
5	ART clinic	Positive	*T. vaginalis*	Insufficient WGS read coverage	0.5	29
6	STI clinic	Unknown	*Neisseria gonorrhoeae*	Insufficient clinical material	ND	ND
7	STI clinic	Unknown	*N. gonorrhoeae*	Insufficient clinical material	ND	ND
*ART, antiretroviral therapy; ID, identification; ND, not determined; STI, sexually transmitted infection; WGS, whole-genome sequencing.						

WGS confirmed LGV (*ompA* sequence identical to those of the *C. trachomatis* L2 434/BU reference strain) in 4 cases with good mean read depth (>12) and high genome coverage (>98%). The 4 sequences clustered well with the L2 sequences previously published and away from L1 and L2b sequences. For 1 specimen, the mean read depth was insufficient to allocate a biovar; insufficient DNA was available from 2 other samples for WGS.

This report shows emergence of *C. trachomatis* biovar L2 genital infection in women living in South Africa, a region to which lymphatic LGV is endemic (*1*). Instead of serologic analysis, we used molecular testing and WGS of clinical specimens to confirm the diagnosis, determine genetic relatedness, and identify the specific variant of genotype L. We observed LGV in specimens from women at the academic hospital in Pretoria, but not from women living in the Mopani District, ≈400 km away. Although the distribution of risk factors may be different, the close relatedness of LGV strains suggests that this might be a localized outbreak of genital *C. trachomatis* L2 infection among women living in Pretoria.

The clinical role of genital *C. trachomatis* biovar L infection in women remains to be determined. Analogous to non-LGV *C. trachomatis* infection in women and rectal LGV in MSM, the clinical spectrum of genital LGV in women may vary from a mucosal ulcer with intrapelvic lymphadenopathy to cervicitis with vaginal discharge, or it may manifest without any symptoms at all as persistent asymptomatic infection. Although rectal *C. trachomatis* infections have been reported in African women, the occurrence of rectal LGV is unknown (*10*).

The emergence of genital LGV in women poses a concern in our setting, which uses syndromic management for STIs, because it is unclear whether the infection would be treated adequately with the empirical regimen of azithromycin and ceftriaxone. The main limitation of this report is the lack of follow-up data to confirm whether syndromic management was effective for these biovar L *C. trachomatis* genital infections.

In conclusion, this report shows the emergence of genital *C. trachomatis* L2 infection in South African women. Further research about its distribution in the general population, clinical role, and the occurrence of rectal infections is warranted because it is unclear whether this STI is managed adequately under the current syndromic management guidelines.
